# Mobile stance-taking in nature: an exploration of gaze patterns during assessments of objects in nature

**DOI:** 10.3389/fpsyg.2024.1461123

**Published:** 2025-02-11

**Authors:** Barbara Laner

**Affiliations:** University of Freiburg, Freiburg im Breisgau, Germany

**Keywords:** assessments, stance-taking, affective stances, gaze, preference organization, walking and talking, mobile interactions, noticing

## Abstract

In this paper, I examine the interactional dynamics of walkers assessing entities in nature, focusing on gaze behavior during these sequences. The analysis is based on a corpus of 10 hiking pairs who walked through the Black Forest National Park while wearing mobile eye-tracking glasses to record their gaze behavior and verbal practices. Using a combined quantitative and qualitative approach, the research identifies gaze patterns in 127 sequences and highlights the role of bodily-visual practices. Contrary to existing literature, the findings indicate that mutual gaze in this setting is not used to mark affiliation but instead occurs only during strong disagreements about initial assessments. During agreements, walkers maintain a triangular position, both gazing at the assessable object without looking at each other. Thus, in this context, gazing at each other serves different interactional purposes, as this study will demonstrate.

## 1 Introduction

Expressing viewpoints and evaluating objects is omnipresent in everyday interaction. Whether we discuss food preferences, exchanging political views, or talking about our favorite movies, we are constantly expressing opinions and evaluating the world around us (*cf.* among others Siromaa and Rauniomaa, 2021: 96). So, it is also common to share stances toward entities in the surrounding environment while experiencing nature together, such as during a walk through the forest (*cf.* Auer et al., 2024; Botsch et al., 2025). Using gazes, gestures, body positions, movements, and language, different aspects in the changing environment can be made relevant. Interactants produce interactional noticings (*cf.* Sacks, 1995; Schegloff, 2007: 87, FN17) whenever they refer to something in nature and make it relevant for the interaction. In the same turn, speakers frequently express their stances toward the noticed object (e.g., look how beautiful that is). Our mutual understanding of the world around us is thus led by sharing observations and discoveries and negotiating evaluative stances toward these.

Research has shown that gaze behavior plays a crucial role during assessment sequences, as “gaze directions and gaze shifts in particular sequential positions in interaction have an important role, first, in relation to one’s right to assess a referent […] and, second, in relation to one’s ability to assess a referent” (Haddington, 2006: 283). By establishing or avoiding eye contact, we can direct attention, signal agreement or disagreement, and even express power dynamics (see discussion below, Section 2.3). Therefore, the study of gaze is an important aspect of analyzing stance-taking in human interactions. Although the importance of gaze behavior is undisputed, most studies focus on non-mobile settings (e.g., Kendrick and Holler, 2017; Siromaa and Rauniomaa, 2021; Pekarek Doehler et al., 2021; Krug, 2025), even though gaze behavior is influenced by moving through space, such as while walking (*cf.* Auer and Laner, 2025).

The activity type I am interested in is joint walks in the forest, where participants need to pay attention to the path in addition to nature and possible assessables, which influences their gaze patterns. I will focus on gaze patterns within sequences in which objects in nature are assessed, i.e., in which evaluative stances are shared. Specifically, I will examine gaze behavior in two parts of the sequence: first, the gaze behavior before and during the first assessment, and second, the gaze behavior when the recipient responds to the assessment. I aim to show that gaze patterns during the first part align with the findings for static interactions described by Haddington (2006, p. 284), but deviate in the second part of the sequence.

In Section 2, I will discuss the theoretical background of the researched activity type, noticings and their cooccurrence with assessments, and findings on gaze behavior during assessment sequences in more detail. Section 3 will provide a methodological overview, before I will present my analysis in Section 4. I will start with a quantitative overview of the data and general findings, followed by a qualitative analysis of gaze patterns during the assessment sequences. The findings will then be discussed in the conclusion in Section 5.

## 2 Background

### 2.1 Walking through nature together

Walking together is not only to be understood as a fundamental form of locomotion but also as a social practice. Social science studies, which have been interested in walking together as “doing walking” since the 1970s (Ryave and Schenkein, 1974: 265), describe this as a joint achievement by the participants and as socially and physically co-organized action. Whenever two people walk together, they usually do so in a side-by-side configuration where walking and stopping, talking, and even gaze must be coordinated. This coordination occurs interactively, and various studies on walking together (Mondada, 2017; Merlino and Mondada, 2019) show that this is a very orderly process and constitutes a social practice characterized by strong mutual “monitoring” (*cf.* Deppermann and Schmitt, 2007: 121; Stukenbrock, 2015: 54).

Building on this understanding of walking as a coordinated social practice, walking through a forest adds additional layers of complexity to interaction. In this natural setting, participants not only need to coordinate their movements and conversations but also navigate the terrain and engage with the surrounding environment. The forest provides a dynamic backdrop where the interplay of nature and social interaction becomes particularly evident. As walkers encounter various natural objects and landmarks, they frequently pause to observe, assess, and discuss these elements, further enriching their joint walking experience.

Joint walks through nature usually do not solely aim to transfer participants efficiently from point A to point B, nor do they exclusively serve as occasions for talk without a directional goal. Walks or hikes in nature typically feature intermittent phases of silence and conversation. Even when not engaged in conversation, walkers are in an “open state of talk” (Goffman, 1981: 134). Additionally, conversations during walks also include both displaced and situated speech. Displaced speech has no topical restrictions^1^, while situated speech during forest walks often refers to environmental features that contribute to spatial orientation, wayfinding, or the shared experience of nature [c.f. for a more detailed discussion Auer et al. (2024)].

Experiencing nature together is an integral part of forest walks (Rauniomaa et al., 2019; Lehmann, 1999; Burckhardt, 2006; Botsch, 2021; Auer et al., 2024; Botsch et al., 2025) and can even be one of the main purposes of going on a hike through the National Park. As a result, these walks centrally involve acts of referring to and discussing noteworthy objects in the surroundings. I am interested in how walkers use verbal and bodily resources to share and express their stances toward certain aspects or objects in nature, with a particular focus on their gaze behavior during these sequences.

### 2.2 Noticing and assessing

The social action of making something relevant in the immediate environment (and calling joint attention to it) has been greatly discussed in literature as the action of ‘doing a noticing’ (*cf.* Auer et al., 2024; Goodwin and Goodwin, 2012; Kääntä, 2014; Keisanen, 2012; Laanesoo and Keevallik, 2017; Laner, 2022; Sacks, 1992, 1995; Schegloff, 1988, 2007; Stivers and Rossano, 2010: 9; Szymanski, 1999). Through an interactional noticing (*cf.* Sacks, 1995; Schegloff, 2007: 87, FN17), an object or phenomenon can be made relevant as noticeable in the immediate environment (Schegloff, 1986; Schegloff and Sacks, 1973). Schegloff (2007: 219) describes this as a “source/outcome” relationship, where the speaker retrospectively reacts to a perceived entity and highlights it as noticeable. From the recipient’s perspective, an interactional noticing stands in a sequentially first position (*cf.* also Keisanen, 2012: 201). However, the social action of “calling joint attention to and achieving intersubjectivity over a selected publicly perceivable referent” (Pillet-Shore, 2020: 7) can also include assessments (*cf.* Golato, 2002; Goodwin and Goodwin, 1986; Goodwin and Goodwin, 1987; Heritage, 2002; Heritage and Raymond, 2005; Pomerantz, 1984; Stivers and Rossano, 2010). As an example, assessments can occur in combination with perception imperatives (e.g., ‘look how beautiful that tree is’, *cf.* also Laner, 2022).

It becomes evident that a clear separation between noticing and assessment is not always possible. Goodwin and Goodwin (2012: 275) also note that an assessment can simultaneously be a noticing. In this paper I will focus on sequences in which assessments occur in sequentially first positions and thereby mostly also function as noticings.

### 2.3 Gaze behavior during assessments

Gaze behavior is a fundamental aspect of human interaction that has garnered considerable attention in interactional linguistics. Its role in social dynamics, particularly during evaluative stance-taking sequences, is multifaceted and pivotal. As asserted by Goodwin (1981), gaze itself can become socially relevant, transforming into a crucial tool for communication. This notion finds further support in the work of Haddington (2006: 299), who argues that mutual gaze expresses convergent stances, underscoring its importance in signaling agreement or alignment during interactions involving evaluation. Agreeing second assessments are preferred, while disagreeing second assessments are dispreferred in most contexts. In more detail, he elucidates distinct functions of gaze in the context of assessments. Firstly, gaze acts as a means for interactants to identify and focus on assessable objects, facilitating the construction of a shared *participation framework* (Goodwin and Goodwin, 2004). This use suggests a visual grounding mechanism that reinforces joint attention and participation in assessing activities. Secondly, it is further argued that mutual gaze between two interlocutors often accompanies their expressions of agreement regarding an assessable, highlighting gaze as a tool for displaying mutual understanding and like-mindedness, reinforcing social solidarity. Thirdly, speakers integrate gaze with verbal and non-verbal cues to position themselves in relation to the stances proposed by their coparticipants, either affiliating with or diverging from these positions. Finally, listeners interpret coparticipants’ gaze trajectories as cues to understand speakers’ stances, contributing to the ongoing negotiation of meaning and interactional alignment (*cf.* for more detail Haddington, 2006).

Kendrick and Holler (2017: 2) shed further light on the expressive function of gaze, noting its role in moderating the level of arousal and emotionality. They propose that gaze direction in the context of polar questions serves as a resource for constructing affiliative and disaffiliative actions, which aligns with Kidwell’s (2006) and Haddington’s (2006) findings, emphasizing its significance in stance-taking and maintaining social solidarity. The research by Kendrick and Holler (2017) illuminates how gaze patterns contextualize responses, with preferred answers (agreeing second assessments) often accompanied by mutual gaze and dispreferred ones (disagreeing second assessments) met with averted gazes. This dichotomy underscores the nuanced role of gaze in managing conversational preferences and social dynamics. Similar, interactional data from various languages reveals a recurrent multimodal practice that respondents deploy in turn-initial positions in dispreferred responses to actions such as information requests, assessments, proposals, and informing (see Robinson, 2020): This practice involves the verbal delivery of expressions equivalent to the English ‘I do not know’ and its variants, coupled with gaze aversion from the prior speaker (*ibid.*). This ‘multimodal assembly’ serves as a preface to dispreferred responses across various sequence types. The use of ‘I do not know’ combined with gaze aversion is a routinized multimodal resource for prefacing dispreferred responses, extending beyond responses to polar questions and encompassing a wide range of sequence types, including responses to proposals, assessments, and informings (*cf.* Pekarek Doehler et al., 2021). These findings not only align with earlier studies linking gaze aversion with dispreference but also broaden the scope to demonstrate that this practice holds across a diverse set of languages, highlighting the universality and significance of this multimodal interactional strategy.

Pekarek Doehler et al.’s (2021) excerpts further demonstrate that recipients’ gaze aversion from prior speakers typically occurs in the transition space or simultaneously with the response onset. Rarely does it take place during the preceding speaker’s turn, and even then, it happens after the recognition point of the prior action and the conditionally relevant next action. Respondents also tend to return their gaze to the prior speaker toward the end of their responsive turn, marking a shift in engagement. They argue that these behaviors serve various functions: Gaze aversion can project a dispreferred response in a premonitory way, signaling the nature of the upcoming response. Conversely, maintaining gaze on a speaker can indicate an expectation of turn continuation, while mutual gaze between participants often signals alignment, even during moments of disagreement (*ibid.*).

However, Krug, 2025 found that participants use gaze aversion to signal self-involvement as a state of unavailability, managing interactional impasses resulting from disalingment. Krug observed that participants redirect their foveal attention to interactionally less relevant areas to avoid visually addressing other participants. The timing of gaze aversion in Krug’s data aligns with the findings of the aforementioned literature, reinforcing its importance as a communicative practice.

In sum, these findings suggest that gaze aversion is a critical visual practice for displaying disalignment and projecting dispreferred responses. Nonetheless, research on gaze behavior during assessment sequences remains limited, as Krug, 2025 also notes for phases of disalignment, highlighting the need for further investigation. In this regard, Kendrick and Holler (2017: 18) emphasize that mutual gaze is more prevalent in face-to-face configurations, suggesting that spatial arrangements significantly influence gaze dynamics. This observation underscores the importance of considering environmental factors in understanding gaze behavior and its implications for social interaction.

Regarding gaze behavior in mobile settings, various studies have shown that speakers naturally spend less time gazing at each other compared to static settings (Auer and Laner, 2025; Laner, 2022; Auer and Zima, 2021; Stukenbrock and Dao, 2019). Thus, gaze is not the primary means to establish a sense of togetherness (“with,” Goffman, 1971), but rather the synchronized moving together through space while walking alongside each other. Nevertheless, gaze remains an important interactional resource for response mobilization and monitoring, such as during question-answer sequences or sequences that include *laughables* (*cf.* Auer and Laner, 2025), meaning that participants do look at each other frequently, albeit for very brief periods, resulting in a low average duration of mutual gaze.

In summary, gaze behavior plays a multifaceted role in evaluative stance-taking sequences, influencing social dynamics and communication patterns. While much progress has been made in understanding its functions and implications, there remains ample room for further exploration, particularly in different settings (and different number of participants, as Rühlemann et al., 2019 propose). By delving deeper into the complexities of gaze behavior, researchers can enhance our understanding of human interaction and pave the way for more nuanced analyses of social dynamics. In this regard, the current study adds to this by exploring a setting that has not received much attention in stance-taking research, namely joint walks that occur mainly in side-by-side formations. This formation has also been described by Kendon (1990); however, gaze, and especially mutual gaze, naturally differ from vis-à-vis settings. For a more detailed discussion, see Auer and Laner (2025).

## 3 Data and method

The current contribution is based on ten videos from a corpus that was created within the research project Looking, Noticing, Talking: How Walkers Experience the Black Forest National Park^2^ that consists of twelve video recordings, each approximately 90 min long. Participants walked with mobile eye-tracking glasses along a pre-determined route through the Black Forest National Park. Specific orientation points were given to the hiking pairs beforehand for navigation. The glasses allow for a precise analysis of the participants’ gaze behavior, which is essential to reconstruct how joint attention is established and how objects in nature are noticed and evaluated. For sequence analysis, methods of interactional linguistics (Couper-Kuhlen and Selting, 2001, 2018) and multimodal conversation analysis (e.g., Mondada, 2014; Sidnell and Stivers, 2013) were applied.

**Figure 1:**
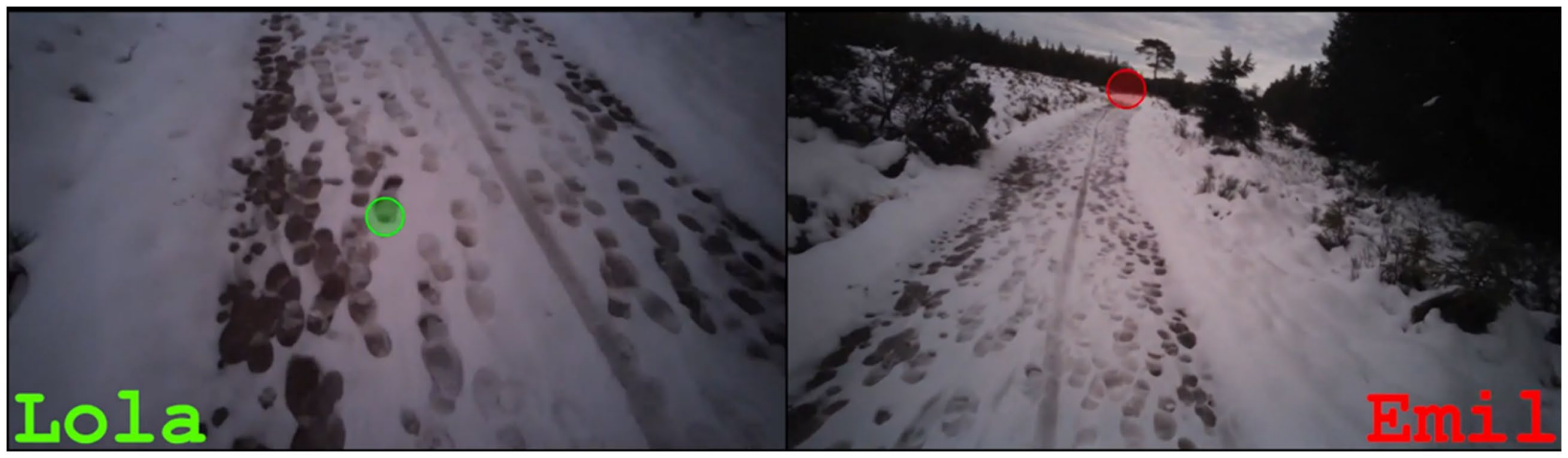
Split screen of the synchronized data.

To collect the present data, mobile eye-tracking glasses from Tobii^3^ were used, which record both gaze behavior and conversations. An external camera was not used in order to create a more natural recording situation. After collection, the data from the hiking pairs were anonymized, synchronized, and split screens were created using Adobe Premiere Pro^4^, in which the recordings of the two hikers are displayed side-by-side (see Figure 1). The red and green dots in the split screen show where the participants are currently looking. However, it must be noted that the front camera of the eye-tracking glasses does not cover the entire human field of vision (for a detailed discussion on the method, see Weiß, 2020: 38–43).

**Figure 2:**
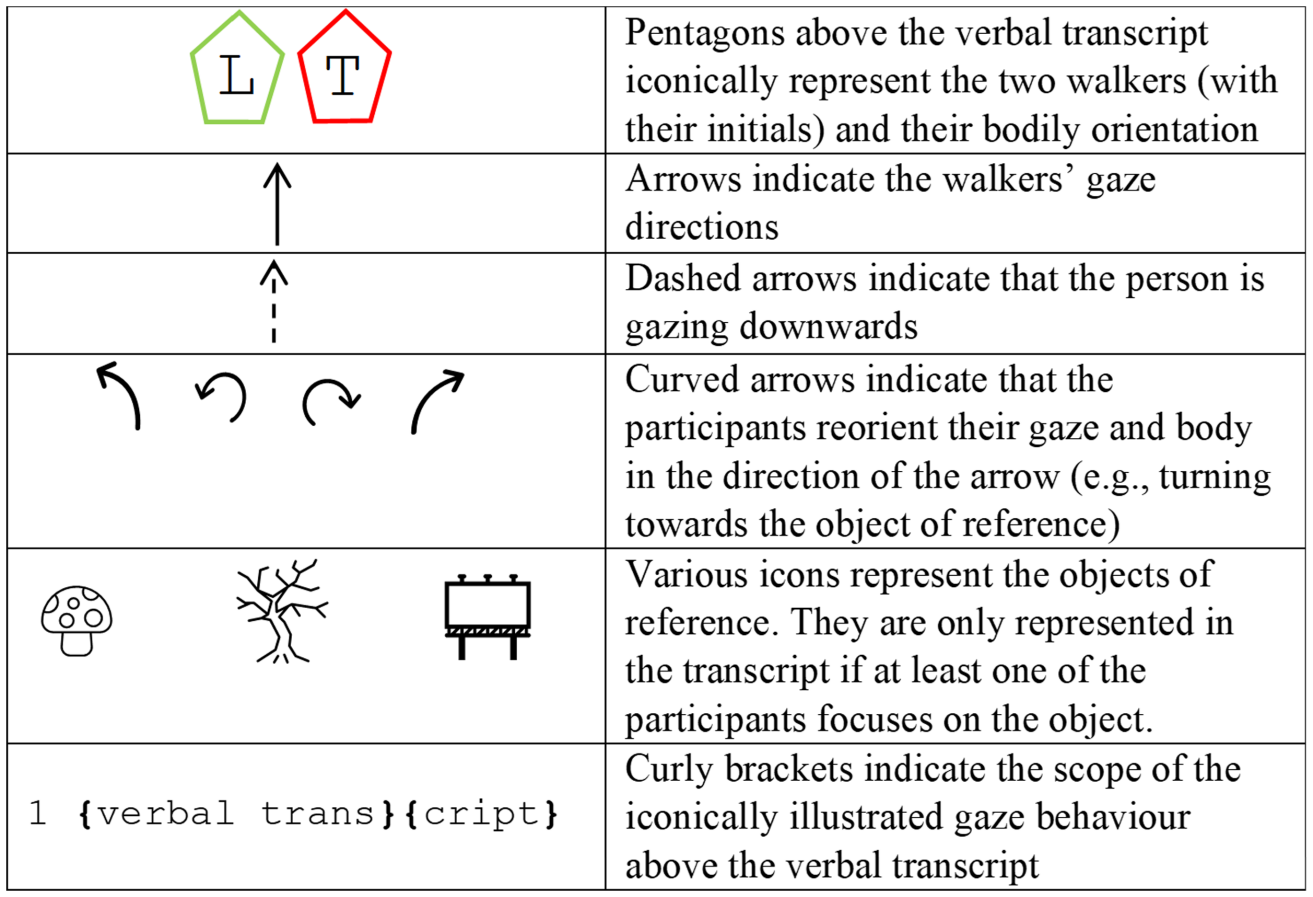
Symbols used for gaze transcription.

In a next step, all instances of assessments that occur in sequentially first positions were extracted and transcribed following GAT2 (Selting et al., 2009). The data excerpts used in this publication were further enhanced with still images and detailed non-verbal information, following the approach of Mondada (2017) and Merlino and Mondada (2019). Additional symbols above the verbal transcript illustrate the bodily orientation and gaze behavior of the interlocutors (see Figure 2), enabling a comprehensive multimodal analysis. For a detailed description of the conventions for the transcription of gaze *cf.* Laner (2022).

## 4 Analysis

### 4.1 Assessments in joint walks

In the current dataset, there are 98 instances of first assessments containing evaluative adjectives^5^ in first positions that refer to entities in the surroundings, along with another 29 cases of general assessments of the surroundings (e.g., evaluating nature in general or commenting on the weather). Thus, assessments in the first position occur 127 times in the data during situated speech. Conversely, only 19 cases of assessments in first position can be found in the data during displaced speech. The study will focus on the cases during situated speech but will briefly discuss first assessments in displaced speech in Section 4.4 for comparison.

First assessments in the data occur in various formats (see below), including exclamatives (*cf.* in detail Auer et al., 2024). They may be prefaced for example by German perception imperatives ‘guck’ or ‘schau’ (both meaning ‘look’), and/or interjections (*cf.* response cries Goffman, 1978), which can serve additional interactional functions (e.g., guiding the other’s gaze or implicitly expressing affective stances before doing so explicitly). All the examples provided are extracted from the corpus and are produced as firsts. I will focus on the gaze patterns within these sequences.

- Oah kUck mal wie **SCHÖN**. (‚oah look PTCL how beautiful’)
- !OH! SCHAU mal-= =ein **schÖner** FLIEgenpilz. (‚oh look PTCL a beautiful toadstool’).
- **GEIL** die wurzeln wo die han; (‚sick the roots that they have‘)
- **TOLL** wie das Alles so (.) verMOOSt is auch; [ne?] (‚awesome how this is all full of moss, right?’)
- dA **schöner** WEIHnachtsbaum, (‚there pretty christmas tree‘)
- sieht so **CRAzy** aus. (‚looks so crazy‘)
- die BANK is **sÜß** da; (‚the bench is cute there’)
- der BAUM is **cOOL**. (‚the tree is cool‘)
- der schaut A **schön** aus; ge? (‘that one also looks beautiful, right?’)

**Figure 3:**
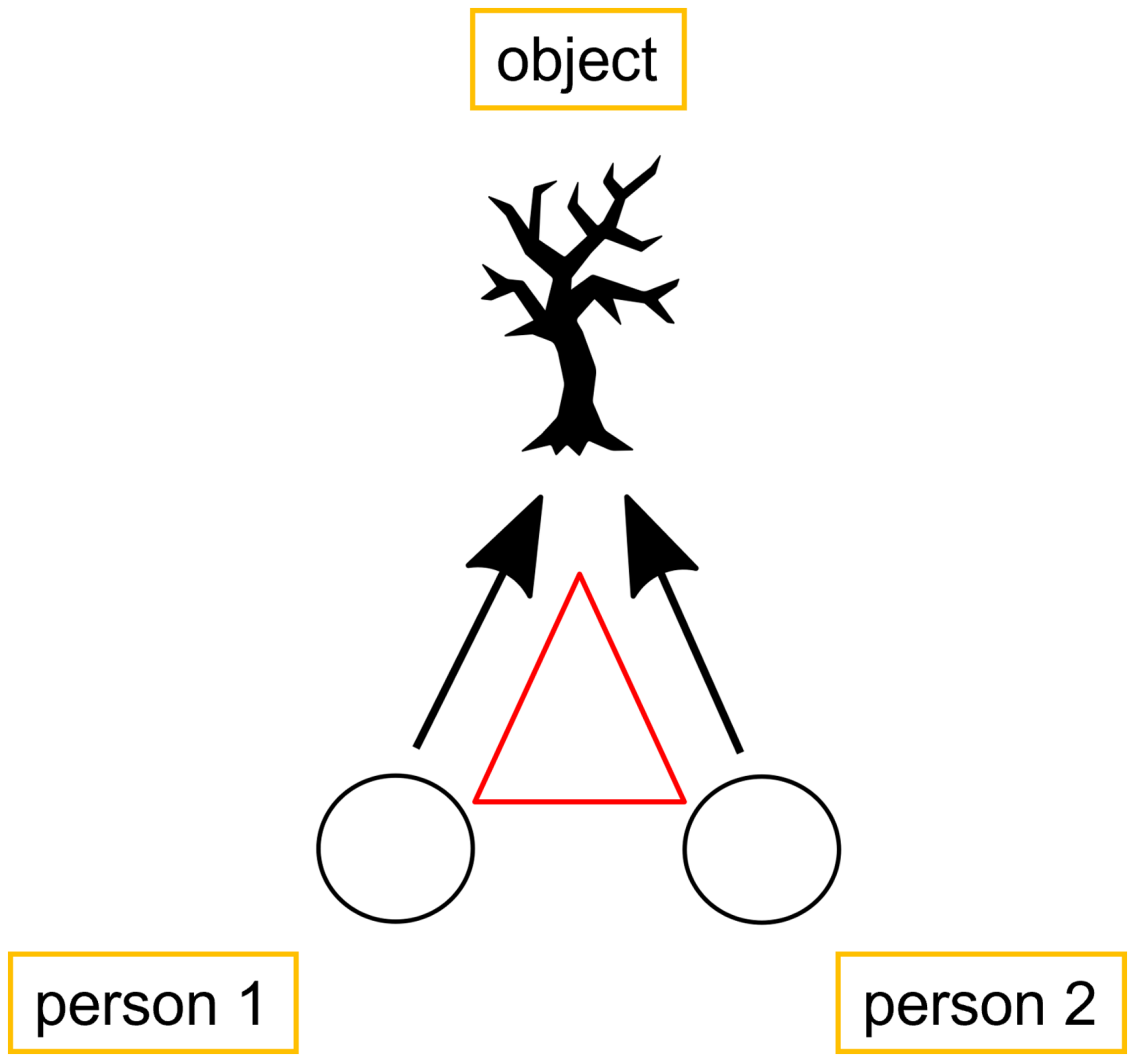
Participants gaze at an assessable in nature.

The discussed literature in the background section suggests that there are two phases that become important for analyzing gaze behavior during assessment sequence: (1) the phase in which participants establish joint attention and one participant assesses the referent and (2) the phase in which the other participant responds to this first assessment. The stance triangle (*cf.* Du Bois, 2007: 163 and in this collection *cf.* de Vries et al., 2024) that is often discussed in literature can be applied to the current data as illustrated in Figure 3.

As shown, participants typically position themselves in a triangular formation toward the assessable. For the first part of the sequence, they adopt and maintain this position during the establishment of joint attention and the first assessment. During the second part (responding to the assessment), it is suggested that participants either turn toward each other and engage in mutual gaze (when showing agreement, *cf.* Haddington, 2006) or avert their gaze (when disagreeing with the initial assessment, *cf.* Robinson, 2020). This may be because, upon hearing an assessment, the recipient turns its gaze toward the person who made the initial assessment to show agreement. Consequently, Haddington (2006) argues that the participants are likely to look at each other during (agreeing) second assessments.

**Figure 4:**
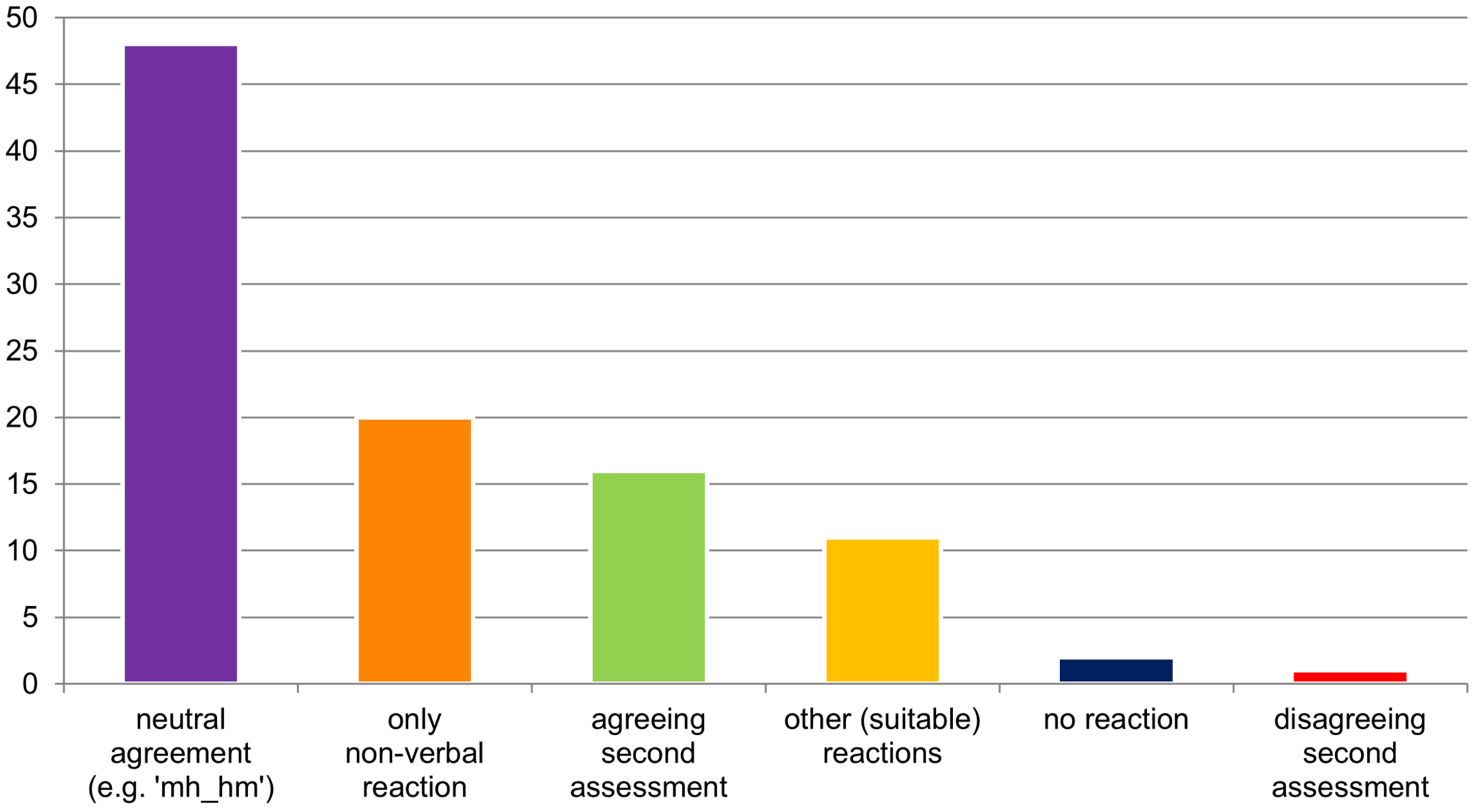
Distribution of different reactions to the assessments.

The gaze patterns in the data align with those described by Haddington (2006: 284) for the first part, including instances where walkers evaluate objects, they are both already looking at (see also Botsch et al., 2025). However, gaze behavior deviates from Haddington’s observations in the second part, which becomes particularly evident in cases of disaffiliation as discussed in section 4.3.1. Note at this point that Haddington’s findings are derived from static settings, where participants are seated around a table. This may be one factor influencing the differences observed. However, in the current context, the first part of the interaction typically occurs while participants are still walking, whereas the second part can take place during a stationary phase – i.e., when participants have stopped in front of the assessable, either during or after the noticing-assessment. This means that, in cases where participants halt to inspect a noticed object in nature and possibly agree or disagree with the initial assessment, there is little difference from stationary settings during this phase, as they can easily look at each other. Before delving into these gaze patterns, I will first provide an overview of how participants verbally react to initial assessments. Almost every second assessment is responded to in a very short and relatively neutral form (see Figure 4), such as with a simple “yes/yeah” or “mh_hm” (*cf.* acknowledgement tokens; Jefferson, 1984). Another 20% of assessments receive only non-verbal reactions, such as gaze and bodily positioning toward the assessed object^6^ (*cf.* also Extract 5). Contrary to expectations, only about one-sixth of the reactions are formulated as second assessments (approximately 16%, see above), with only one case contradicting the initial assessment.

Another way to react to the initial part of the assessing sequence is not to directly respond to the assessment itself, but to the object by asking more about it or expressing surprise upon seeing it (e.g., a rare plant). These cases are categorized as ‘other (suitable) reactions.’ For the sake of completeness, there are also two cases in which no reaction to the assessment follows. In these sequences, something else captures attention immediately after or almost simultaneously with the assessment, resulting in the assessment being neglected.

Altogether, it becomes clear that what is assessed as ‘beautiful,’ ‘nice,’ ‘cool,’ or ‘sad’ in nature is almost always agreed upon. This is likely due to cultural and social conventions that loosely define what is considered esthetic and what is not. This cultural imprinting, combined with the familiarity among participants, explains why disaffiliation – i.e. disagreement on the initial assessment – rarely occurs in the data.

### 4.2 Gaze patterns during affiliating assessments of objects in nature

As already discussed, Haddington (2006: 309) argues that participants first look at an assessable while one of them utters a first assessment and subsequently establish mutual gaze whenever the other participant adopts a convergent stance about the assessable. The act of jointly looking at the assessable typically begins before the actual assessment is uttered and is maintained for a significant period. In contrast, mutual gaze, which Haddington (2006) describes as occurring during the second part of the assessing sequence to display a convergent stance in response to the initial assessment, does not occur in the discussed data during a second assessment.

The activity of jointly looking at the assessable can be observed in each assessing sequence in the data. In the following section I will discuss two examples in which language is needed to introduce a new focus on an object that is assessed subsequently and two in which something is assessed that is already more or less in the focus of the participants, showing differences in the choice of utterances (the findings are consistent with Auer et al.’s (2024) findings for the use of how-exclamatives with or without a preceding perception imperative). In each case, the gaze behavior in the second part of the assessing sequence will be discussed in detail, too.

#### 4.2.1 Establishing joint attention and assessing an object in nature subsequently

In the majority of cases (61%), one participant gazes at an object before calling attention to it and assessing it. This happens very quickly and precisely, rather than in a slow manner where joint attention is established separately from the assessment sequence. Eye-tracking data, which records gaze behavior precisely, shows that this process occurs sequentially and in an orderly fashion. In 21 cases, a perception imperative introduces the assessment. These imperatives can be uttered in its own intonation phrase or within the same phrase as the assessment, typically following one another without pauses in between. In all these cases, the gaze reaction usually begins immediately after the perception imperative is uttered (for a detailed discussion see Laner, 2022: 11). The following two excerpts illustrate this.

In the first example, the two walkers, Anke and Iris, are just concluding a conversation about a hike in Corsica. Following this sequence, attention is drawn to a small cave on the left side of the path. The small pentagons iconically represent the physical orientation of the participants, and the gaze behavior is depicted by arrows.

**Extract (1): Little Cave** (#Kleine Höhle; VP2122)


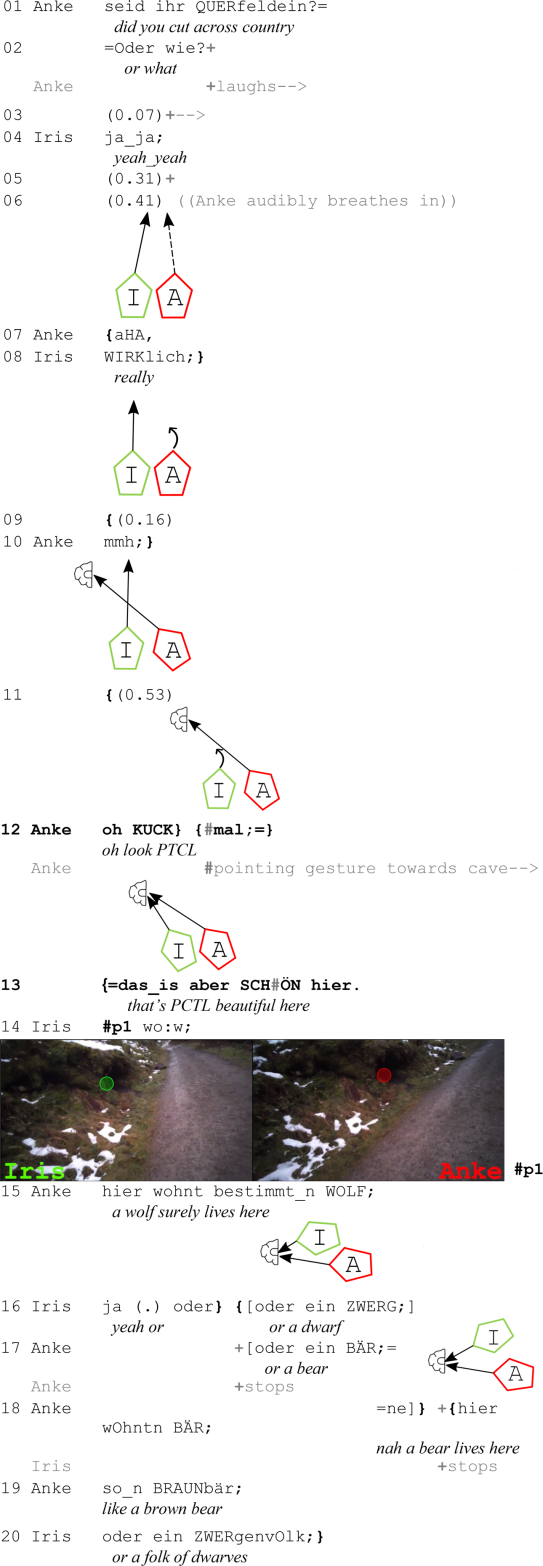


Toward the end of the preceding sequence, during which the two walkers talk about a previous hike, Anke turns to the left side of the path (line 09), with the gaze point from her eye tracking glasses showing that her gaze is directed at a small cave in line 11. A good half-second later, she then produces “oh KUCK mal;==das_is aber SCHÖN hier.” (*oh LOOK PTCL== this is PTCL NICE here*; lines 12–13). Here, the perception imperative is preceded by the response cry ‘oh’ (*cf.* Goffman, 1978; Golato, 2012; Anna and Pfeiffer, 2021; Pfeiffer and Anna, 2021), which enhances the affective stance that is then made explicit through the assessment ‘this is PTCL NICE here’. The addressee’s bodily reaction occurs very quickly: Right after ‘look’ in line 12 and simultaneously with Anke’s pointing gesture, Iris starts to refocus toward the space of reference. By the beginning of the assessment in line 13, her gaze is already directed at the targeted object. Thus, immediately after uttering the perception imperative (line 12), joint attention is established. Soon after, Iris lowers her hand and ends the pointing gesture. This occurs just before the end of her assessment in line 13.

As exemplified in the first part of this assessment sequence, the refocusing by the addressee (here line 12) typically begins before the place and/or object of reference is even mentioned or specified. Recipients, therefore, anticipate in this setting that ‘look’ is used in its literal sense (i.e., not as discourse marker, see Deppermann, 2021: 201, 203 and Günthner, 2017: 105) and they routinely align their gazes at the entity of reference. The effectiveness of this can be partially explained by the strong mutual monitoring (particularly through peripheral vision) that occurs while walking together (Deppermann and Schmitt, 2007: 121; Stukenbrock, 2015: 54), allowing participants to infer where the speaker is most likely referring to (for a more detailed discussion on the usage of perception imperatives in German see Laner, 2022).

For the second part of this sequence, Iris verbally reacts to the assessment with “wow” (line 14), indirectly acknowledging that she has recognized the object of reference and affiliating herself with Anke’s positive assessment (on alignment and affiliation in more detail see, among others, Stivers, 2008; Steensig, 2019). During this phase, they both continue to gaze at the assessable and even afterwards. There is not a single gaze toward each other, even though they have stopped to inspect the little cave. This suggests that, even though they have entered a stationary phase – comparable to stationary settings as observed in Haddington (2006), Kendrick and Holler (2017), and Pekarek Doehler et al. (2021) – they do not establish mutual gaze during the affiliating second assessment. Only Haddington included assessments of objects in the current surroundings, though, which results into building up a stance triangle (Du Bois, 2007: 103) with a visible object as shown in Figure 3.

In the second example, the two participants are in an open state of talk, before one of the walkers starts to point at a tree on the right side of their hiking trail and assessing it.

**Extract (2): Tree** (#Baum; VP2728)


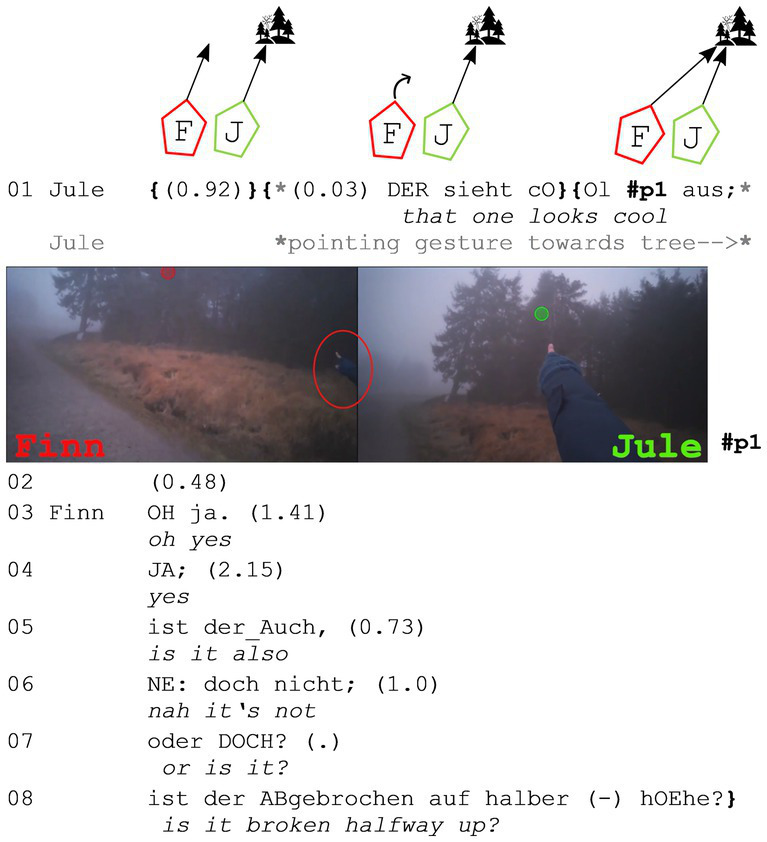


In this case, there is no perception imperative preceding the assessment, but a pointing gesture begins before the utterance, which is rare in the data (they usually start later). As seen in the still from Finn’s camera (#p1, left side), the pointing gesture is clearly visible in his right periphery. This likely explains his early gaze reaction (turning toward the object) at the start of Jule’s assessment (line 1). When Jule utters her assessing adjective “cool” (line 1), both are already gazing at the assessable. After a short pause (line 2), Finn reacts with “oh ja” (*oh yes*, line 3), agreeing with her assessment of the tree. They then discuss whether the tree is broken at medium height, but this part of the discussion is excluded for reasons of space.

As in extract (1), joint attention is established right before the assessment is made explicit (“schön,” *beautiful* in extract 1 and “cool” in extract 2). Thus, the same gaze pattern can be observed during the first part of the sequence. The reactions in these two sequences differ in that the first is responded to with a second assessment (“wow”), whereas the second is responded to with “oh ja” (*oh yes*), which also conveys (affective) agreement (see Golato, 2012 for a discussion of “oh” as an affective change-of-state token). Despite the slight difference in reactions, no difference in gaze behavior is observed in the second part of the sequence: Both participants only gaze at the assessable and never at each other. In the next part, I will discuss two examples in which the gaze behavior in the first part of the sequence differs (a joint focus on the assessable is already established before the sequence with the assessment even begins).

#### 4.2.2 Assessing an object in nature that is already in the focus of both walkers

As Botsch et al. (2025) argue, joint attention (or at least a joint focus) can be inferred by two walkers from their bodily behavior. This becomes especially clear when both walkers stop (more or less together) to gaze at an object in nature. Here again, a subsequently ordered gaze pattern can be observed, but for the first part, no verbal exchange is needed: Both participants orient toward the later assessed object and gaze at it (and stop to do so, if it is not an object far ahead). In the example below, the two participants are talking about a town nearby when they both stop and gaze at the course of a small stream.

**Extract (3): Streamlet** (#Bachlauf_1; VP0506)


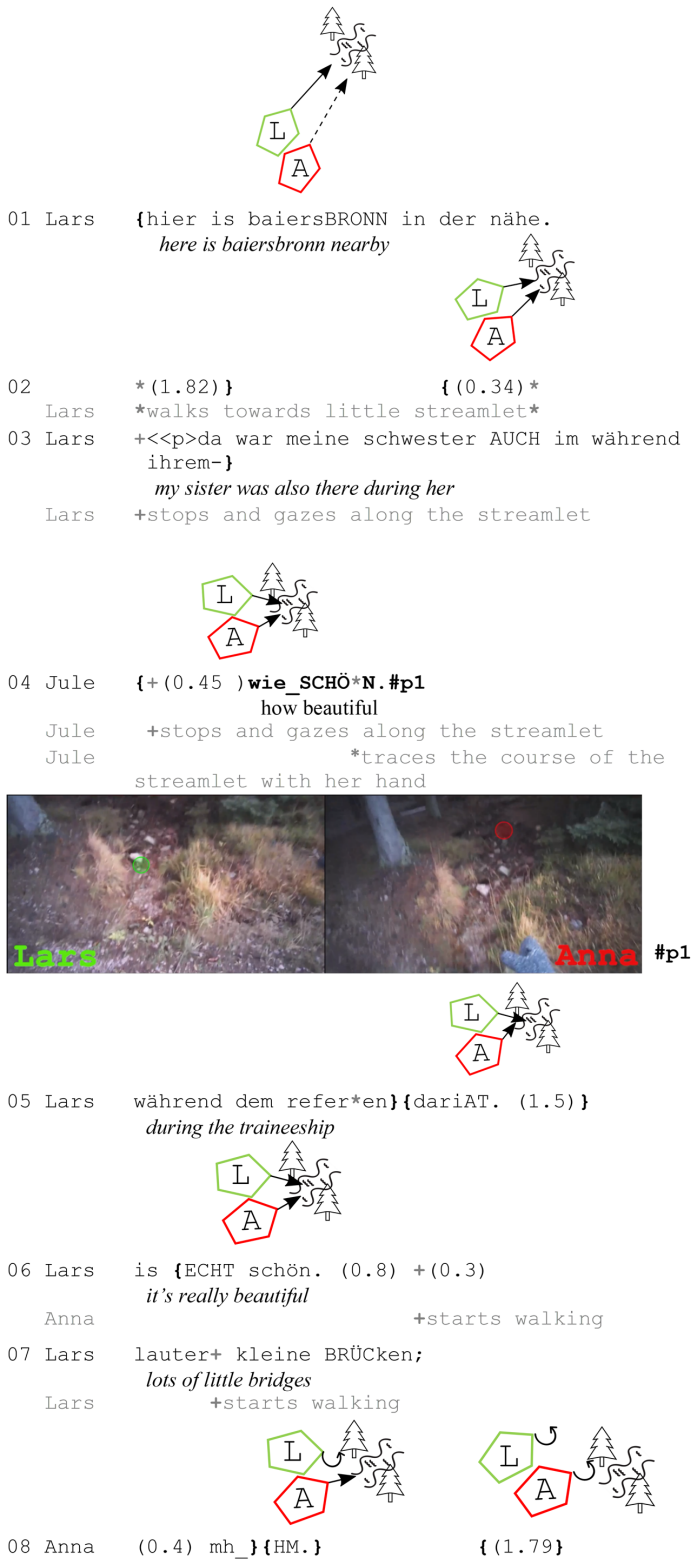


While Lars makes his reference to Baiersbronn in line 1, he starts to gaze at a small stream and its course. Shortly after (line 2), Anna also gazes at it. Both stop subsequently (Lars in line 3 and Anna in line 4) before Anna assesses it with “wie SCHÖN” (*how beautiful*, line 4). As both walkers have stopped and are gazing at the streamlet, it can be assumed that they are both aware that they are looking at the same object [*cf.* Botsch et al. (2025)]. “Wie schön” (*how beautiful*) here can be described as a minimal form of a how-exclamative (see Pfeiffer, 2017: 43). The reduced form of “wie” (*how*) and the evaluative adjective “schön” (*beautiful*) strongly supports the assumption that both participants have already accomplished a common visual focus that is presupposed by both of them [as argued by Auer et al. (2024): 269f, extract 6].

The observable gaze pattern during the first part of the assessment sequence is in this case similar to the one seen in the first two extracts, but without verbal exchanges being necessary. Although joint attention is already established, Anna initiates an iconic pointing gesture toward the end of her assessment, tracing the course of the streamlet. This gesture highlights that she finds the course of the streamlet beautiful, not just the streamlet itself.

Lars finishes his utterance about his sister in line 5 before he reacts to Anna’s assessment with an upgrade: “is ECHT schön” (*it’s really beautiful*, line 6). Here again, the two walkers stay in their triangular position facing the assessable without once glancing at each other.

In the next example, both walkers are also gazing at the assessable before the assessing sequence starts. In contrast to the example before, the participants do not stop in this extract, but continue to walk while they talk about the moss that can be seen everywhere on the left side of the walking trail.

**Extract (4): Moss** (#Moosstämme; VP1920)


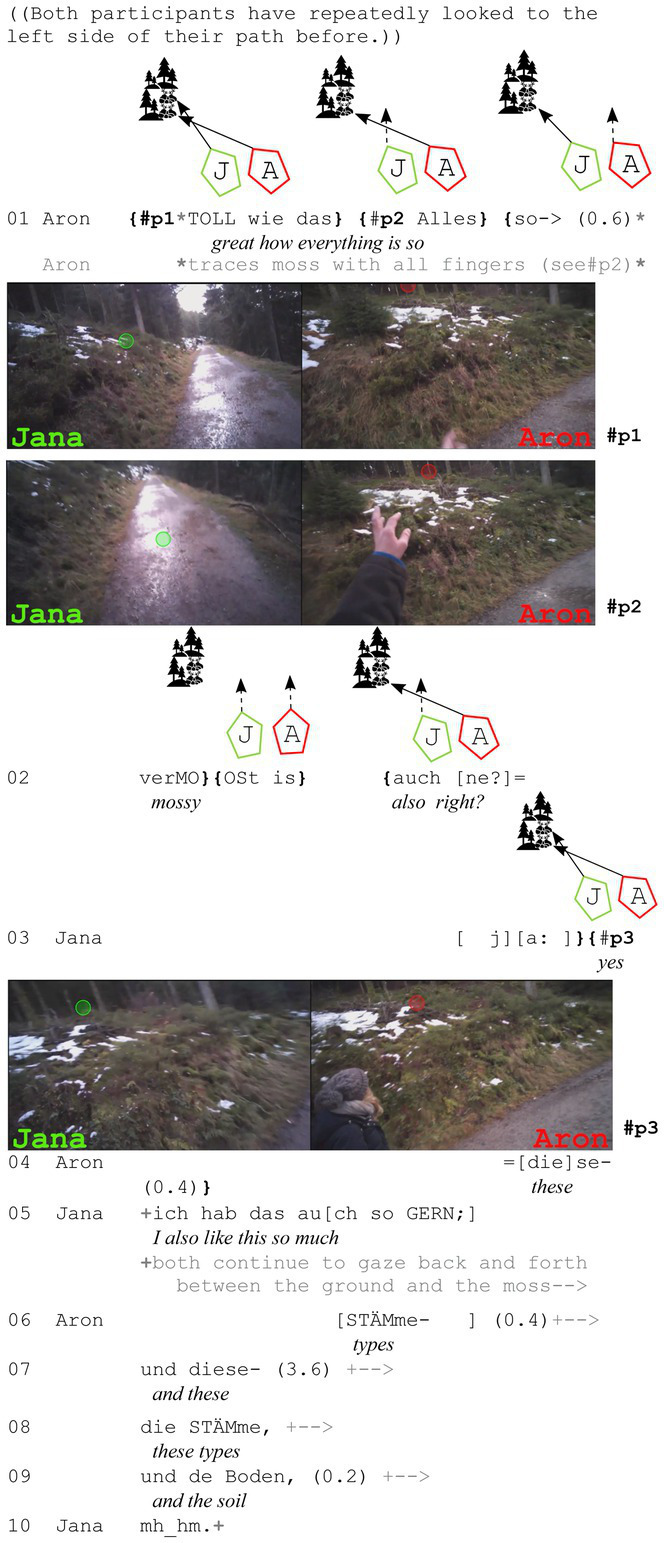


In this case, it is important to emphasize the bodily positions of the walkers. Jana walks on the right side and because of Aron’s orientation toward the left side, she is now even more perceivable in his periphery. Thus, he can perceive that she is also looking at the left side and seems to infer she is also gazing at the moss on this side of the path, since he neither produces any perception imperatives nor uses any initial (local) deictic terms. He starts with an assessing adjective^7^, but still uses an iconic gesture (see #p1) – as in the example before – to illustrate his point. Jana agrees with “ja” (*yes*) and continues to explain that she likes that a lot too.

In the extracts discussed so far, I have explored the intricate relationship between gaze behavior, joint attention, and assessments in mobile settings, revealing a distinct pattern of gaze use during the noticing-assessment process. The first two examples clearly demonstrate a sequential gaze pattern, where both participants direct their gaze toward the object to be assessed before the assessment is verbally articulated. This pattern is consistent across various extracts, with the only variation being whether the initial gaze occurs before the verbal utterance [extracts 3 and 4, *cf.* Botsch et al. (2025)] or during the noticing-assessment (extracts 1 and 2). Despite this variation in timing, the second part of the assessment sequence remains consistent: participants continue to gaze at the object while agreeing (affiliating) with the initial assessment. This continuity suggests that gaze serves as a fundamental tool for establishing joint attention during the whole noticing-assessment sequence, regardless of when it is initiated and how participants react to it.

This observation is crucial because it challenges assumptions about the role of gaze in signaling affiliation. In many other contexts, mutual gaze is an important marker for agreement (or affiliation), as seen in the work of Haddington (2006), Kidwell (2006), Kendrick and Holler (2017), and Pekarek Doehler et al. (2021) among others. These studies highlight how mutual gaze can serve to visibly signal a convergent stance and mutual agreement. However, in the current setting of mobile side-by-side interactions, we find a significant departure from this pattern. Contrary to what might be expected based on previous research, walkers in this study do not engage in mutual gaze to signal their agreement with the other participant’s assessment. Rather, gaze is used primarily to establish joint attention, not to indicate affiliation.

This raises the question: do gazes toward each other simply not occur during mobile side-by-side interactions, or do they serve a different function altogether? As will be shown in the following section, participants do indeed gaze at each other in these contexts, but for reasons that differ significantly from signaling affiliation and agreement. This distinction is important, as it suggests that gaze serves a more fundamental role in ensuring attention is directed toward the object of assessment, rather than fostering mutual agreement between participants.

Drawing on Haddington’s (2006) work, we can understand this pattern more clearly. Haddington argues that participants first look at an assessable while one of them makes an initial assessment, and mutual gaze typically emerges during the second part of the assessing sequence, when the other participant adopts a convergent stance toward the object. In this sense, mutual gaze functions as a sign of affiliation, showing that both participants are in agreement with the assessment. However, in the data discussed here, mutual gaze does not occur during the second agreeing assessment. Despite having entered a stationary phase, which could be compared to the stationary settings described by Haddington (2006), Kendrick and Holler (2017), and Pekarek Doehler et al. (2021), participants do not establish mutual gaze during the second assessment.

This divergence suggests that gaze behavior during mobile interactions functions differently than in stationary settings. While mutual gaze may be used to display affiliation in stationary settings, in mobile contexts, it serves more to establish joint attention on the object being assessed, rather than signaling agreement between the participants themselves. This shift in function highlights the adaptive nature of gaze in different interactional settings, where one of its roles in one context – such as signaling affiliation – may change in another context, where its role is more focused on directing attention to the object of assessment.

Moreover, only Haddington’s study included assessments of objects in the immediate surroundings, which contributes to the formation of a stance triangle (Du Bois, 2007: 103) involving the two participants and the object that is assessed. This triangle (Figure 3), is critical to understanding the unique nature of gaze behavior in these contexts. The presence of a visible object in the current surroundings plays a central role in the interaction, influencing how participants engage with it and each other. While mutual gaze may emerge in stationary settings as a way of displaying affiliation, in mobile settings, it seems that gaze is less concerned with signaling agreement and more focused on ensuring that both participants share attention toward the object being assessed – even during stationary phases, whenever participants have stopped in front of the assessable as in extracts 1 and 3.

In conclusion, these findings suggest that gaze behavior in mobile interactions functions primarily as a tool for establishing joint attention rather than signaling affiliation. This insight challenges previous assumptions about the role of gaze in social interaction and calls for a more nuanced understanding of how gaze operates across different interactional settings. By focusing on the ways in which gaze facilitates shared attention to the object of assessment, we gain a deeper appreciation of its role in mobile social contexts, where the dynamics of interaction differ from those in stationary settings (*cf.* also Auer and Laner, 2025). Further research is needed to explore the subtleties of gaze behavior in mobile interactions and to better understand how gaze functions in other contexts than the typically investigated sitting arrangements.

### 4.3 Gazing toward each other during assessment sequences

As shown in the extracts before, assessment sequences that proceed in a regular manner, i.e., in a side-by-side configuration and with preferred reactions to the first assessment, the two walkers never gaze at each other. However, there are seven cases in the data in which participants gaze at each other. In this section I will first show an example in which the speaker of the assessment gazes at its recipient, before I will show an example in which the recipient of the assessment gazes at the speaker. I will then conclude in the last section (4.3.1) with an extract in which both walkers gaze at each other, i.e., establish mutual gaze.

In the following example, the two walkers are in an open state of talk when one of them perceives a toad stool on the right side of the path which will be assessed in the following.

**Extract (5): Toad stool** (#Fliegenpilz5_1; VP0506)


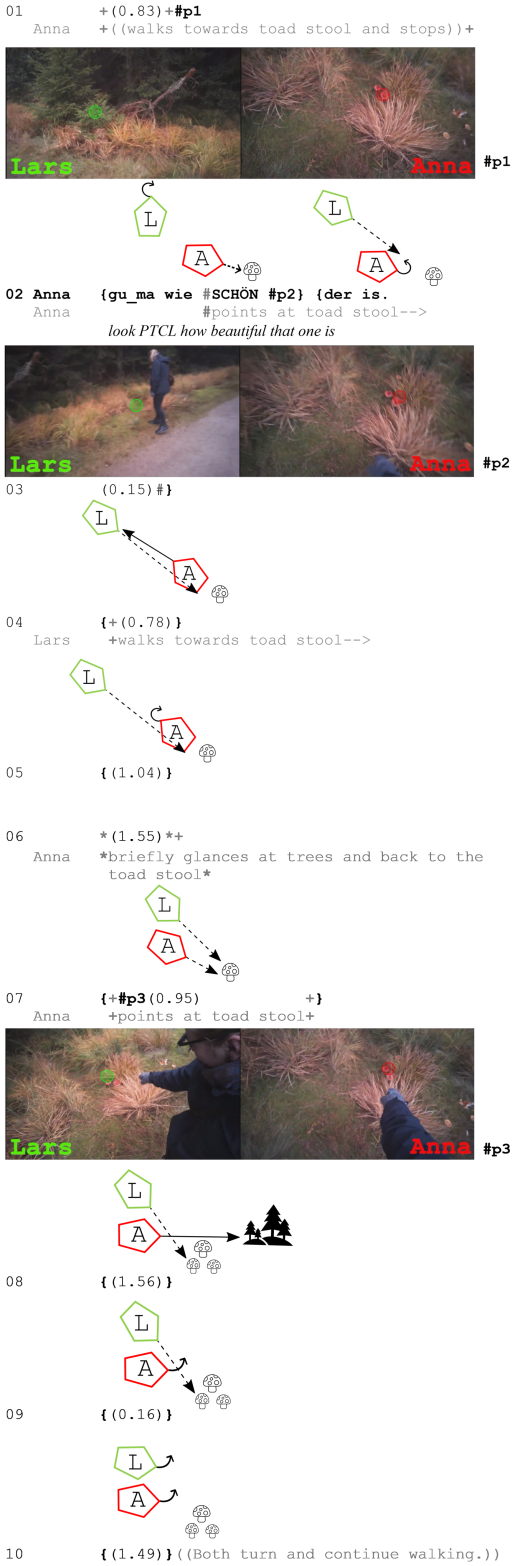


Before Anna assesses the toadstool as beautiful, she first walks toward it and stops right in front of it. This behavior has two consequences: First, she has moved away from her walking partner (she has fallen behind) and therefore cannot perceive him in her periphery anymore (she has even turned her head completely away from Lars, as can be seen in #p2). Second, Lars seems to recognize that she has stopped and fallen behind because he already starts to turn around at the beginning of line 2, right before she starts her utterance. He follows her gaze and looks at the assessable shortly afterward (also line 2). However, toward the end of Anna’s assessment, she turns completely around to look at Lars. She gazes at Lars during line 4, when he starts to walk (back) toward her and the toadstool. She then turns back to the assessable again (line 5) and waits for him while briefly gazing at the trees and back to the toad stool (line 6). In line 7, she presumably perceives that he is now next to her in her periphery, because she then leans forward and produces another pointing gesture (#p3). They both continue to gaze at the toadstool before subsequently turning and starting to move again together.

In this example, the speaker of the assessment gazes toward her recipient. This happens already during the uttering of the assessment and only in cases in which the participants have separated from each other or the referent is especially hard to ‘detect’ and a lot of direct monitoring and explaining is needed to show the other the assessable. Cases like these are rare (only 4 cases in the data set), but they show that participants do look at each other during assessments, but for other reasons than affiliation – in this case the monitoring function of gaze is crucial.

The next extract shows one of the two cases in the data in which the recipient of the assessment gazes toward the speaker (so the other way around to the case before). As in the extract before, the two participants are in an open state of talk before the assessment sequence.

**Extract (6): Foggy Nature** (#Natur_Nebel; VP0304)


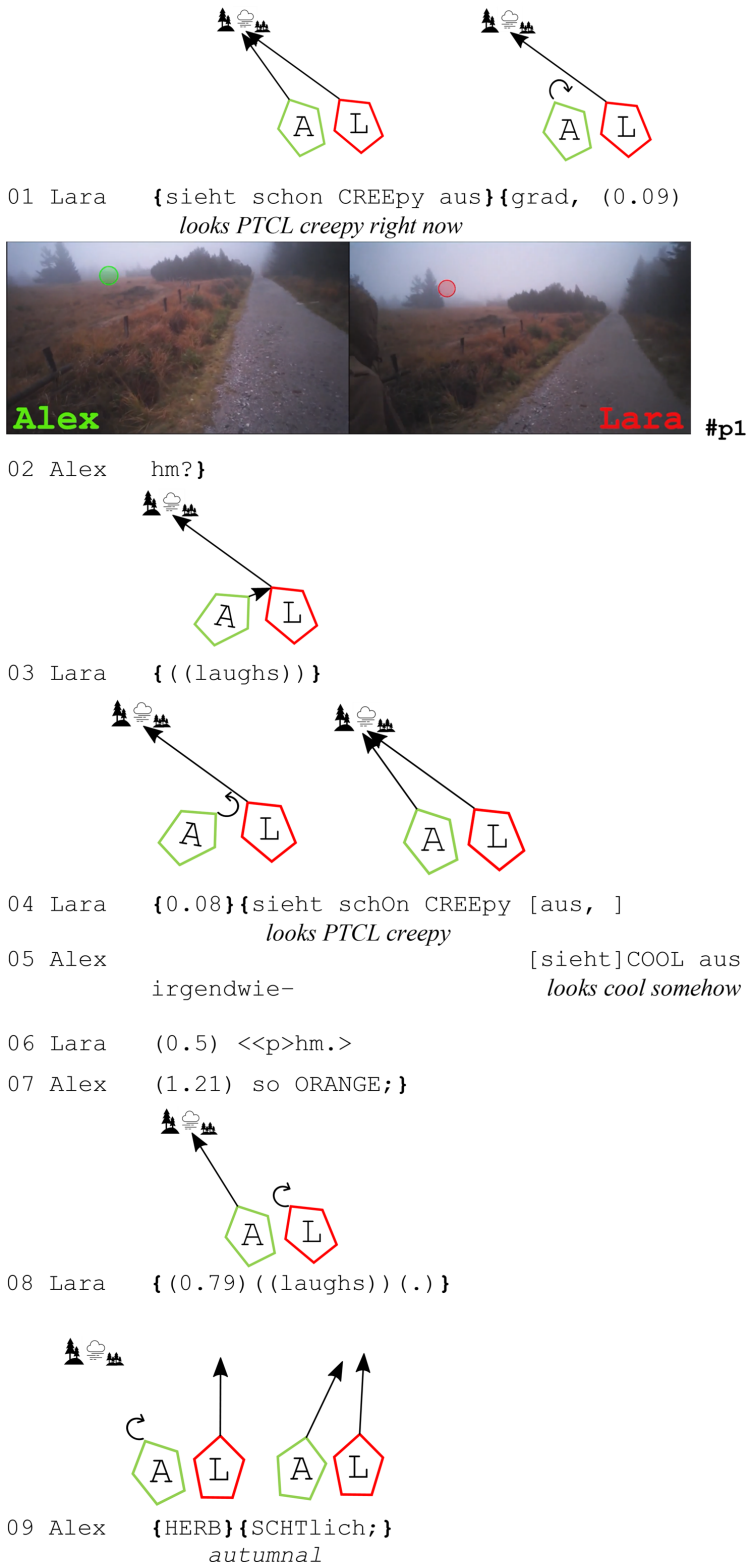


Here, both participants gaze at the landscape covered in fog before Lara utters, “sieht schon CREEpy aus grad” (*looks pretty creepy right now*, line 1). Alex does not understand her and initiates repair with “hm?” in line 2. She then laughs and repeats her assessment of the foggy landscape (lines 3 and 4). During her repeated assessment, Alex assesses the nature as ‘somehow cool’ (line 5) because the leaves are “orange” (line 7), and it looks “herbstlich” (*autumnal*, line 9). Although at first glance it might seem that the assessments diverge, both assess slightly different aspects of what they are gazing at in this moment, but still show agreement (e.g., Lara utters “hm” in line 6). Additionally, they have repeatedly talked about this during their hike that the foggy landscape looks a bit ‘creepy’ but also ‘cool’ at the same time.

Here, a gaze toward the speaker of the first assessment can be observed. This happens during the repair initiation and thus apparently is not connected to the assessment itself. This gaze behavior during repair initiations is also found in other sequences in the data that do not contain assessments.

As these two extracts (5 and 6) have shown, whenever one of the participants gazes toward the other, it happens for monitoring reasons (speaker toward recipient, extract 5) or while initiating repair (recipient toward speaker, extract 6). These gaze orientations toward the other walker are not connected to the assessment sequence but have different interactional reasons. However, there is one case in which participants turn toward each other and even establish mutual gaze because of what happens during the assessment sequence. This will be discussed in the following subsection, 4.3.1.

In the previous chapter, I observed that gaze behavior during assessment sequences primarily serves to establish joint attention to the assessable object rather than signaling affiliation between participants. Mutual gaze, a typical marker of agreement or convergence in other contexts (Haddington, 2006; Kendrick and Holler, 2017; Pekarek Doehler et al., 2021), was notably absent during the assessment process, particularly during the second part of the assessing sequence, when participants are expected to agree with the initial assessment. Instead, the gaze was focused on the object, reflecting the participants’ shared attention to the object of evaluation, rather than engaging in mutual gaze as a sign of affiliative alignment.

As the two extracts (5 and 6) in this section have shown, whenever one of the participants gazes toward the other, it serves monitoring purposes (speaker toward recipient, extract 5) or occurs during repair initiation (recipient toward speaker, extract 6). These gaze orientations are not connected to the assessment itself but serve different interactional functions. However, there is one case in which mutual gaze occurs as a result of the assessment process. This finding raises important questions about the conditions under which gaze behavior shifts from being a tool for monitoring and joint attention to other functions. It also offers a nuanced contribution to the literature on gaze dynamics in interaction (Haddington, 2006; Kendrick and Holler, 2017; Pekarek Doehler et al., 2021). In this instance, mutual gaze emerges not as a sign of affiliation, but rather, it is used in the context of strong disagreement with the initial assessment. This provides a rare example of how gaze can shift in response to the content of the assessment. This shift contrasts with earlier findings, where gaze toward each other was not part of disaffiliating second assessments, underscoring the complex, context-dependent nature of gaze behavior in mobile interactions.

#### 4.3.1 Mutual gaze during assessment sequences

As mentioned before, it is very rare for walkers to disagree on how nature is assessed, and it is even more unlikely for them to strongly disagree (see overview in Section 4.1, Figure 4). However, in cases where one walker strongly disagrees with the other’s evaluation of an object, a different gaze pattern emerges during the second part of the sequence. The following excerpt shows this in detail.

In the beginning of this sequence (lines 1–7), they talk about something not connected to nature before one assesses the view (line 8) and subsequently a tree (line 13).

**Extract (7): ‘Crippled tree’** (#Krüppelbaum; VP1516)


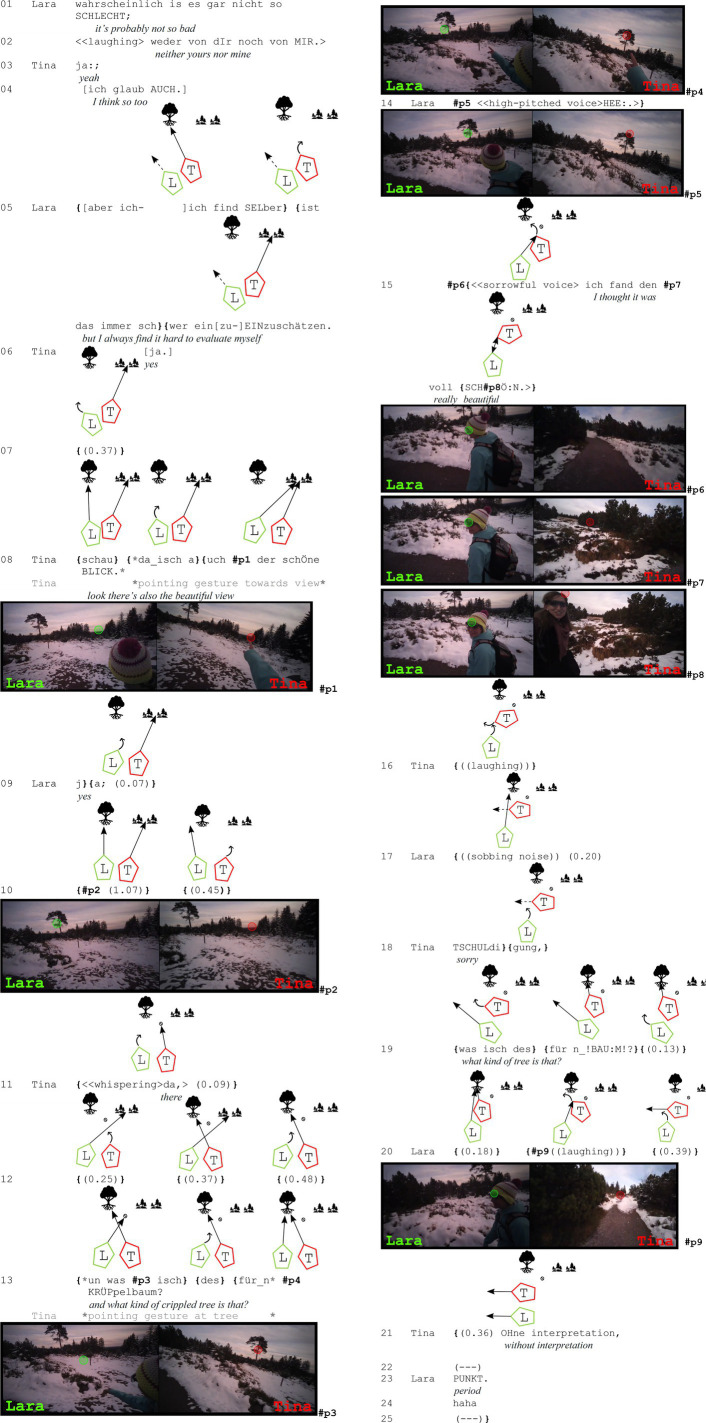


The excerpt above contains two assessments – one of the view and one of a tree. In the first case, Tina assesses the view as beautiful (line 08) and Lara agrees (line 09). Here, they both establish joint attention but do not look at each other (as discussed in the first part of section 4.2). During the second assessment, when Tina expresses her stance toward a tree by evaluating it as ‘crippled’ (line 13), they also establish joint attention: Tina points at the tree and formulates her assessment as a question “un was isch des für_n KRÜPpelbaum?” (*and what kind of crippled tree is that*, line 13). Up until this point, gaze behavior corresponds to the first examples (1 and 2).

Lara reacts with a high pitched “hee:.” (line 14) that sounds almost reproachful and starts gazing at Tina (see #p6). This ‘he’ projects her upcoming disagreeing assessment of the object (similar to prefaces such as ‘I do not know’ or ‘well’, *cf.* among others Robinson, 2020, Pekarek Doehler et al., 2021, and Heritage, 2015). She then utters in a sorrowful voice “ich fand den voll SCHÖN.” (*I thought it was really beautiful*, line 15). During this second assessment, Tina also turns, and they establish mutual gaze at the end of the second assessment (#p8). As they turn away from each other again, Tina laughs (line 16) and Lara mockingly makes sobbing noises (line 17). Then, Tina apologizes (line 18) and reformulates her question, leaving out her negative assessment: “was isch des für_n BAUM?” (*what kind of tree is that?*, line 19) to which Lara reacts with laughter and a brief glance at Tina (line 20). Tina continues with “OHne interpretation” (*without interpretation*, line 21), and Lara concludes with “PUNKT” (*period*, line 23) and laughs again (line 24). Note that all of this takes place while they are walking.

Strongly disagreeing on how an object is assessed can be potentially face-threatening and requires careful negotiation to manage the social dynamics of the interaction. In such cases, participants employ various conversational strategies to mitigate the impact of disagreement as can be seen in this extract: (1) Laughing here serves as a softening mechanism, reducing the tension associated with disagreement and signaling that the disagreement is not meant to be confrontational (line 16, 20, 24). (2) Apologizing as in line 18 can serve to acknowledge the other’s perspective and mitigates the impact of the disagreement. (3) Reformulating (line 19) can further help soften the disagreement, allowing participants to maintain a cooperative atmosphere. On top of all these strategies, mutual gaze (4) seems to play a crucial role in these negotiations, as it facilitates a more immediate and personal connection, enabling participants to gauge each other’s reactions and adjust their responses accordingly. By combining these strategies, participants navigate the complexities of disagreement, maintaining respect and cooperation despite their differing views.

In summary, the last three examples demonstrate that when two people assess things in nature while walking together, they do not simply avoid gazing at each other. Instead, they gaze at each other for specific reasons that are not necessarily connected to the assessment itself. Speakers tend to gaze at recipients when the two walkers are physically distanced from each other, requiring the speaker to monitor the recipient’s position and reactions. Recipients, on the other hand, gaze at speakers during the shown assessment sequences primarily when initiating repair during conversation. Mutual gaze, distinct from these other forms of gaze behavior, occurs due to divergent assessments. When participants strongly disagree, mutual gaze is established as a way to negotiate and resolve the disagreement, likely because such disagreements can pose a face-threatening situation.

### 4.4 Gaze during first assessments in sequences of displaced speech

Although sequences of displaced speech are not the focus of this paper, I would like to briefly address them in this section to provide a broader perspective. Assessments in first position during displaced speech are rare, as they typically occur in second position, e.g., when reacting to a story or something similar. In the current dataset, there are 18 instances of utterances containing assessments in a sequentially first position. Four are used to frame a following story, projecting what kind of story it is, e.g., ‘funny’, ‘sad’, etc. [*cf.* examples (d) and (e)], and two are used to resume a topic previously set aside when participants shifted from displaced to situated speech (e.g., when they noticed something in nature while discussing a topic not related to the current surroundings, *cf.* example f). The other twelve instances are first assessments that introduce a new topic and assess a referent in the same utterance (*cf.* examples a-b) and can either be assessables known to both participants (as in b and c) or not (as in a).

- wir ham da letzte STEINpilze gefunden die war_n richtig geil;
  (‘*we found some porcini mushrooms there last time; they were really awesome*;’)
- <<lachend>die HANna vorhin war au lustig ge,>
  (‘*<<laughing>Hanna was also funny earlier, right,>*’)
- *EOH; (-) KRASS,= =nAEchsten SONNtag ist schon erster advent*;
  (‘*EOH; (-) SICK,= =next Sunday is already the first Advent*;’)
- das war gestern LUStig,=
  (‘*that was funny yesterday*,=)
- äh:m (-) auf der HOCHzeit was_n bisschen n fAIL war-
  (‘*uh:m (-) what was a bit of a fail at the wedding-*’)
- ja also richtig ver[RÜCKT.]
  (‘*yeah, really crazy*.’)

In all these sequences, no differences in gaze behavior were found compared to the cases discussed earlier during situated speech, even though in these instances, no stance triangle (Du Bois, 2007) with a visible object in the surroundings needed to be established – i.e., no joint attention to an entity in nature had to be established. In all these sequences, no differences in gaze behavior were found compared to the cases discussed earlier during situated speech. To illustrate this, I will present two short examples. In the first extract, both walkers are silently walking next to each other before the sequence starts.

**Extract (8): Porcino Mushrooms** (#Steinpilze; VP0102)


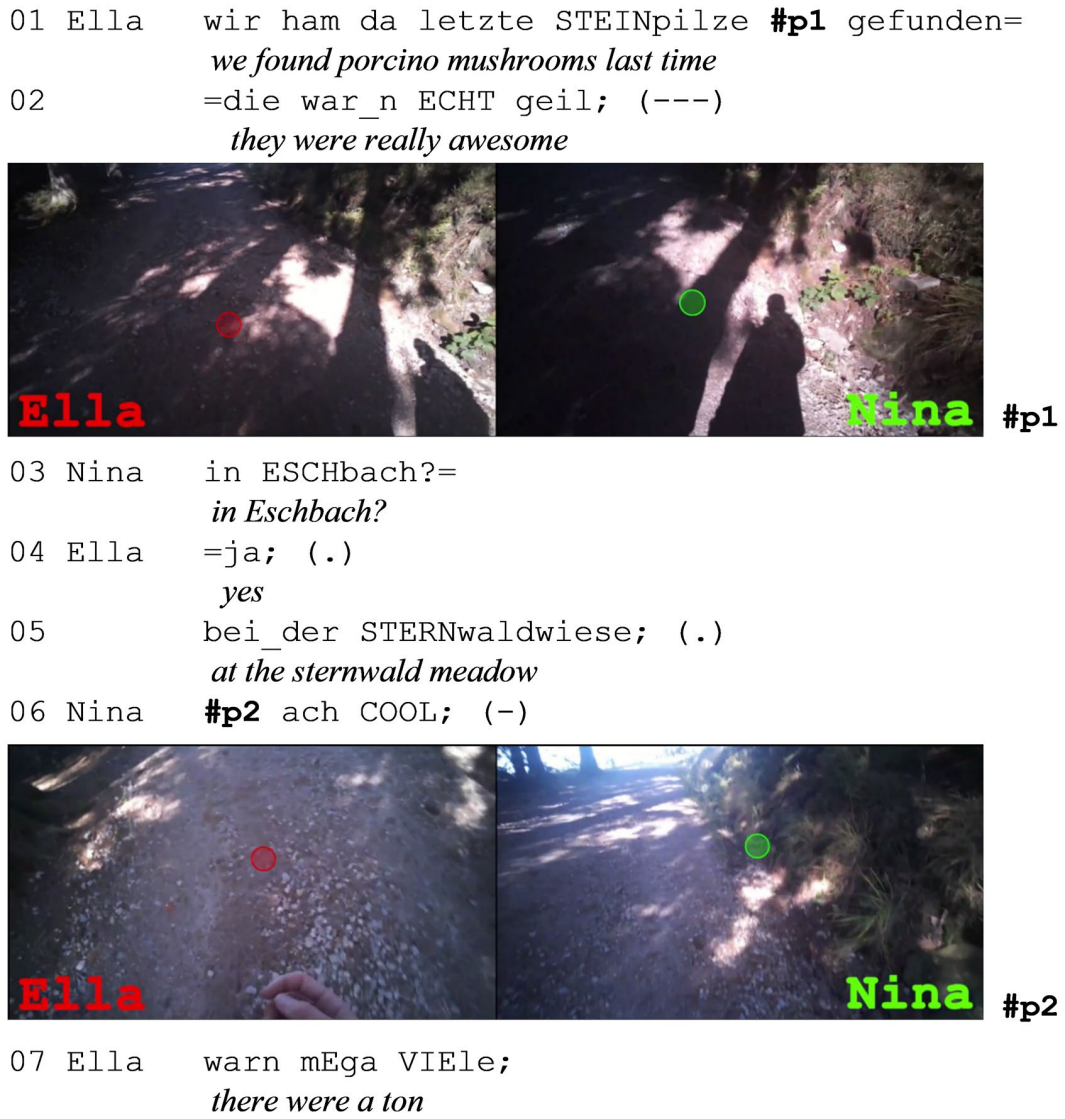


The shown sequence starts with Ella’s utterance in line 1 and 2 that she has found porcino mushrooms which were “ECHT geil” (*really awesome*, line 2). Nina asks subsequently if she found them in Eschbach, which Ella affirms (line 4) and specifies with “bei_der STERNwaldwiese” (*at the sternwald meadow*, line 5). Only then Nina reacts with a second assessment (line 6), which does not assess the mushrooms itself, but the fact that Ella found them. The sequence ends with Ella saying that there were “a ton” of them there (line 7). Here, the assessable that Ella refers to is not accessible to Nina and can therefore only be assessed by Ella. Meaning that only she has the right to assess it (as only she has knowledge about it), which goes hand in hand with only her being able to assess it. Still, Nina produces a second assessment, but refers to the finding of them in Eschbach.

During this short assessment sequence, both participants continue to walk and gaze down at the ground (as in #p1). Their gazes do not remain fixed on one point but wander around the path they are walking on (compare #p1 and #p2). There are no gazes toward each other, and there is no triangular positioning since the assessable is not visible in the current surroundings. Similarly to the cases during situated speech, there are generally no gazes toward each other. However, some cases do involve gazes toward each other, but again for different interactional reasons, as I will shortly show in the last extract.

In the last extract, both participants are again in an open state of talk, before the sequence starts. Here, one participant assesses something that both have access to, i.e., something they experienced together.

**Extract (9): Rope up** (#Anseilen; VP0910)


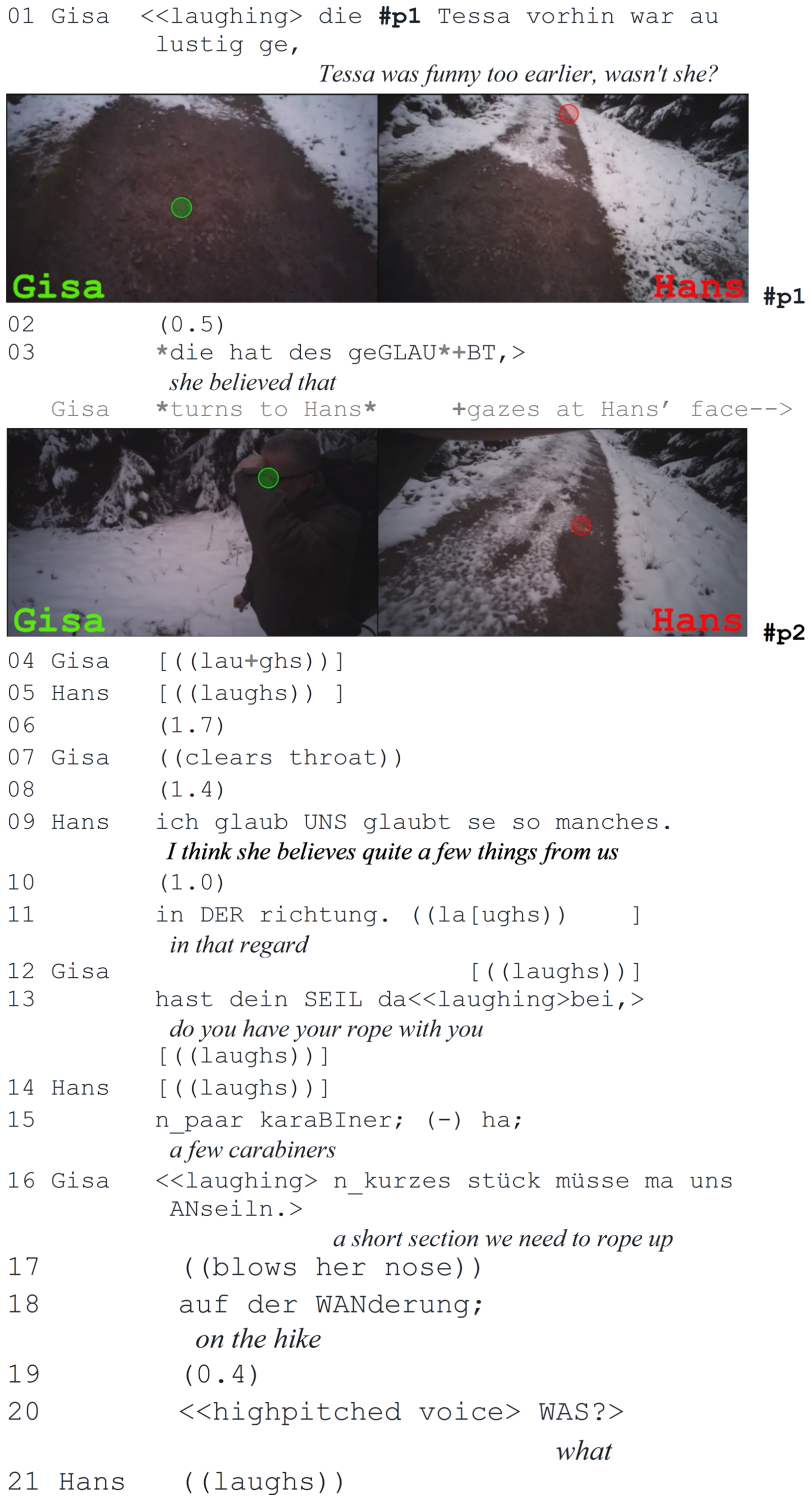


In this sequence, Gisa recalls a funny joint experience, namely that they pulled Tessa’s (a friend of their daughter) leg, by telling her that they would have to rope up during their hike today. Her assessment of Tessa is accompanied by laughter and ends with a question tag (line 1). Similar to findings for “ne” in German, here “ge” (both can roughly be translated with ‘right’/‘wasn’t it’ etc.) is used to indicate that Gisa does not claim exclusive rights to the assessment, showing that they have equal or shared epistemic rights to assess the referent (*cf.* König, 2017: 245). Then there is a short pause in line 2, before Gisa continues with “die hat des geglaubt” (*she believed that*, line 3) and turns toward Hans. Since the joint experience is also a laughable, the gaze behavior falls in line with Auer and Laner’s (2025) findings that speakers regularly gaze at the recipients of their laughable during joint walks. She stops gazing at Hans right after he starts to laugh too.

During the assessment, both participants continue to gaze toward the path they are walking on, as in the previous extract (extract 8). There is only a speaker gaze later that seems to elicit a response, as the gaze stops immediately after the recipient starts to react with laughter.

In this section, the focus was on instances where the assessable is not a visible object in nature, but rather referents that are not present, such as people or objects that are not immediately visible. These cases, although similar to the findings from Kendrick and Holler (2017), Pekarek Doehler et al. (2021), and Siromaa and Rauniomaa (2021), differ primarily in terms of mobility. While the general verbal exchanges are (better) comparable to these studies, as there are no visible referents, the gaze behavior still differs and may be due to the mobility of the participants during the interaction.

However, as demonstrated by Auer and Laner (2025), gazing toward each other does occur during displaced speech, but for different interactional reasons. In contrast to findings in previous studies where mutual gaze often indicates agreement or shared stances (Haddington, 2006; Kendrick and Holler, 2017), in the case of displaced speech, gaze is not used to display affiliation. Instead, it serves different interactional functions, primarily eliciting responses from the other participant. During situated speech, gaze toward each other was similarly not tied to the assessment itself or used to indicate affiliation. Rather, gaze in these contexts functions more as a tool for managing the flow of interaction, establishing joint attention and ensuring that the recipient is engaged and responsive.

Thus, while gaze plays an important role in both types of speech, it does not serve the affiliative function often described in stationary settings. Instead, it is mobilized to manage the interaction and to elicit responses, underlining the complex, context-dependent nature of gaze behavior in dynamic, mobile settings. This distinction between gaze as a tool for joint attention and gaze as a tool for signaling affiliation highlights the different interactional demands at play when assessing not only visible but also displaced referents.

## 5 Conclusion

The present study examined interactions among people walking together to understand the gaze patterns during assessments of entities in the current perceptual space. The goal was to use a conversation-analytic and qualitative approach to identify and explore gaze patterns in each assessment sequence found in the dataset and providing a quantitative overview of these findings. Consistent with existing research focused on interactions while walking, particular importance was placed on bodily-visual practices in the examined sequences, with a special focus on gaze behavior. This study addresses a niche area of research, adding insights into the dynamics of mobile interactions. The conversation-analytic and qualitative approach allows for a detailed understanding of how walkers navigate the dual tasks of walking (and stopping) together while responding to and evaluating environmental stimuli.

Researching this particular setting has revealed that, contrary to existing literature, mutual gaze is not used to mark a “convergent stance” (Haddington, 2006: 299). Instead, the opposite was observed: walkers gaze at each other when they strongly disagree on an assessment of an object in nature. This highlights the social aspect of gaze, as strong disagreement is a face-threatening act (with laughter also playing a mitigating role, as seen in Extract 7). Since there was only one strong disagreement found in the data (although 127 cases of assessments occurred and were investigated), further research is needed to strengthen this finding. Still, the fact that in all the cases where participants agree, the described triangular position was not only held during the first part of the sequence but also during the second part (the response), strongly suggests that the established mutual gaze during the one case of disaffiliation is significant. Furthermore, the establishment of mutual gaze happens while the participants continue to walk, whereas gazing toward each other would presumably be easier if they had stopped, as they did in Extracts (1), (3) and (5).

Thus, in the context of walking together through nature, gaze does not serve as a tool to signal affiliation during assessment sequences. Instead, the primary role of gaze in this setting is to establish and maintain joint attention on the surrounding environment and to ensure safe navigation along the path. The need to focus on the terrain and potential obstacles necessitates that participants direct their gaze toward the path, reducing opportunities for mutual gaze. This practical consideration overrides some of the communicative function of gaze seen in other settings, where for example mutual gaze can signal affiliation or alignment. Consequently, during walking interactions, joint attention to the environment becomes more critical, with participants using their gaze to visually engage with their surroundings rather than each other. This shift underscores that in such mobile side-by-side interactions, the coordination of physical movement and shared environmental focus takes precedence over (some) functions of gaze typically observed in more stationary contexts (and vis-à-vis). It is important to stress, though, that not all functions of gaze known from other settings are different, as seen in extract 6, where one participant initiated repair. In situated speech sequences establishing joint attention is presumably the most important function of gaze (also to monitor each other as in extract 5). During displaced speech, joint attention is not a factor, and therefore other functions of gaze can be found, as Auer and Laner (2025) show.

In addition to these primary findings, the study revealed several notable side findings. Almost half of the assessments were reacted to with short and rather neutral verbal reactions such as “mh_hm” or a simple “yes.” Even more remarkable, 20% of the cases were reacted to only non-verbally, as shown in Extract (5), where a clear reaction is demonstrated by gazing at and walking back to the assessable – but there was still no verbal reaction. Contrary to expectations, second assessments occurred in only 16% of cases. These findings, apart from gaze behavior, are peculiar and presumably connected to the specific setting of the study. Naturally, in a setting where assessments occur so frequently (mean = 14.1 per hike), not every assessment can lead to longer sequences, but is rather quickly acknowledged before continuing to walk. Either way, further research in comparable settings (such as walks in different contexts) is needed to show whether these findings only apply to walks through nature, or whether mobility is the important factor.

In conclusion, mutual gaze does not play a significant role in assessments during mobile side-by-side interactions. Only one instance found in the data is (somehow) related to assessments: mutual gaze during disagreeing second assessments. This behavior indicates that mutual gaze is employed as a strategy to navigate potentially face-threatening moments and negotiate diverging stances. However, further research is necessary to determine if engaging in mutual gaze during mobile interactions is consistently linked to disagreement and face-threatening situations. Understanding these nuances can provide deeper insights into the intricate dynamics of gaze behavior in mobile contexts and its broader implications for social interactions.

## Data Availability

The datasets presented in this article are not readily available because written consent by participants was given to the storage and usage of the data at the University of Freiburg and publications, but not for providing the raw data (the videos) for others. Requests to access the datasets should be directed to barbara.laner@germanistik.uni-freiburg.de or peter.auer@germanistik.uni-freiburg.de.

## Footnotes

^1^*cf.* Auer and Laner (2025) on laughables that are produced during displaced speech in forest walks.

^2^Cooperation of the Black Forest National Park, Department 3 (Dr. Kerstin Botsch, Dr. Susanne Berzborn), and the Chair of German Linguistics at the Albert Ludwig University of Freiburg (Prof. Dr. Dr. h.c. Peter Auer).

^3^Tobii Pro Glasses 2: https://www.tobiipro.com/de/produkte/tobii-pro-glasses-2/.

^4^Adobe Premiere Pro v14.0: https://www.adobe.com/products/premiere.html.

^5^Instances in which stances towards natural objects were shared only implicitly (e.g., through laughter, etc.) are excluded from this investigation.

^6^Note that very small additional non-verbal cues, such as facial gestures (e.g., smiling), might be visible to the speaker peripherally but will not be recorded by the eye-tracking glasses. However, these still fall into this category since there is no language involved.

^7^Note that this structure of utterance (assessing adjective in first position) only occurs in the data when entities in nature are assessed that are not one singular object (e.g., snow, forest, moss).
